# Dynamics behind disjunct distribution, hotspot-edge refugia, and discordant RADseq/mtDNA variability: insights from the Emei mustache toad

**DOI:** 10.1186/s12862-020-01675-8

**Published:** 2020-08-28

**Authors:** Yuchi Zheng, Qiang Dai, Xianguang Guo, Xiaomao Zeng

**Affiliations:** 1 Chengdu Institute of Biology, Chinese Academy of Sciences, #9 of Section 4, Ren-Min-Nan Road, Wuhou District, Chengdu, 610041 Sichuan Province China; 2 Key Laboratory of Southwest China Wildlife Resources Conservation (Ministry of Education), China West Normal University, Nanchong, 637009 Sichuan Province China

**Keywords:** Sichuan Basin, *Leptobrachium boringii*, Disjunct distribution, Climate stability, Last interglacial period, Biodiversity hotspot, Refugium, RADseq, Genetic diversity

## Abstract

**Background:**

The distribution of genetic diversity and the underlying processes are important for conservation planning but are unknown for most species and have not been well studied in many regions. In East Asia, the Sichuan Basin and surrounding mountains constitute an understudied region that exhibits a “ring” of high species richness overlapping the eastern edge of the global biodiversity hotspot Mountains of Southwest China. We examine the distributional history and genetic diversification of the Emei mustache toad *Leptobrachium boringii*, a typical “ring” element characterized by disjunct ranges in the mountains, by integrating time-calibrated gene tree, genetic variability, individual-level clustering, inference of population splitting and mixing from allele frequencies, and paleoclimatic suitability modeling.

**Results:**

The results reveal extensive range dynamics, including secondary contact after long-term isolation via westward dispersal accompanied by variability loss. They allow the proposal of a model that combines recurrent contractions caused by Quaternary climatic changes and some failed expansions under suitable conditions for explaining the shared disjunct distribution pattern. Providing exceptional low-elevation habitats in the hotspot area, the eastern edge harbors both long-term refugial and young immigrant populations. This finding and a synthesis of evidence from other taxa demonstrate that a certain contributor to biodiversity, one that preserves and receives low-elevation elements of the east in this case, can be significant for only a particular part of a hotspot. By clarifying the low variability of these refugial populations, we show that discordant mitochondrial estimates of diversity can be obtained for populations that experienced admixture, which would have unlikely left proportional immigrant alleles for each locus.

**Conclusions:**

Dispersal after long-term isolation can explain much of the spatial distribution of genetic diversity in this species, while secondary contact and long-term persistence do not guarantee a large variation. The model for the formation of disjunct ranges may apply to many other taxa isolated in the mountains surrounding the Sichuan Basin. Furthermore, this study provides insights into the heterogeneous nature of hotspots and discordant variability obtained from genome-wide and mitochondrial data.

## Background

Understanding the spatial distribution of genetic diversity within species and the underlying processes is important for conservation [1–3]. Quaternary climatic oscillations have deeply affected the distribution and genetic diversity of a variety of organisms, especially in the Northern Hemisphere [4, 5]. The resulting relationship between distributional history and diversity is often conditional. For instance, long-term refugia may maintain populations with large effective sizes and high genetic variation, yet drift and inbreeding can cause a reduction in diversity when the refugial areas are small [4, 6]. Alternatively, range expansions are usually characterized by reduced diversity due to the founder effect and the fact that only a portion of all diversity is involved in the expansion [7, 8], while admixture results in increased levels of variation in areas of secondary contact [9]. Such complexities may complicate the prediction of genetic diversity based on the duration of occupation or other measures of unevenness across the geographic range of a species.

Regional biogeographic studies have often identified different major processes that shape the patterns of intraspecific genetic variability, for example, evolutionary melting pots [9, 10], historical climate stability [11–13], and reduction of diversity during dispersal [14, 15]. In relatively well-studied areas, commonalities may emerge regarding the relationship between the patterns of the distributional history and genetic diversity. On the Iberian Peninsula, for different plants and animals, genetically highly divergent populations have persisted in different glacial refugia throughout the Pleistocene; in some of these species, postglacial expansion has occurred and led to less diverse populations at northern latitudes [16, 17]. Such generalizations not only facilitate the understanding of biodiversity patterns but may also provide the empirical basis for predicting the genetic diversity pattern of a species from the inferred distributional history in corresponding regions [11, 18, 19]. Currently, for most organisms, genetic diversity characterization is missing or incomplete. Numerous phylogeographic studies conducted before the widespread availability of high-throughput sequencing used a limited number of loci (often one), and obtained measures of genetic diversity, such as heterozygosity, that depend strongly on a large number of loci [20, 21]. In understudied, species-rich regions, performing phylogeographic studies with comprehensive sampling should be one of the first steps in understanding and conserving biodiversity.

**Fig. 1:**
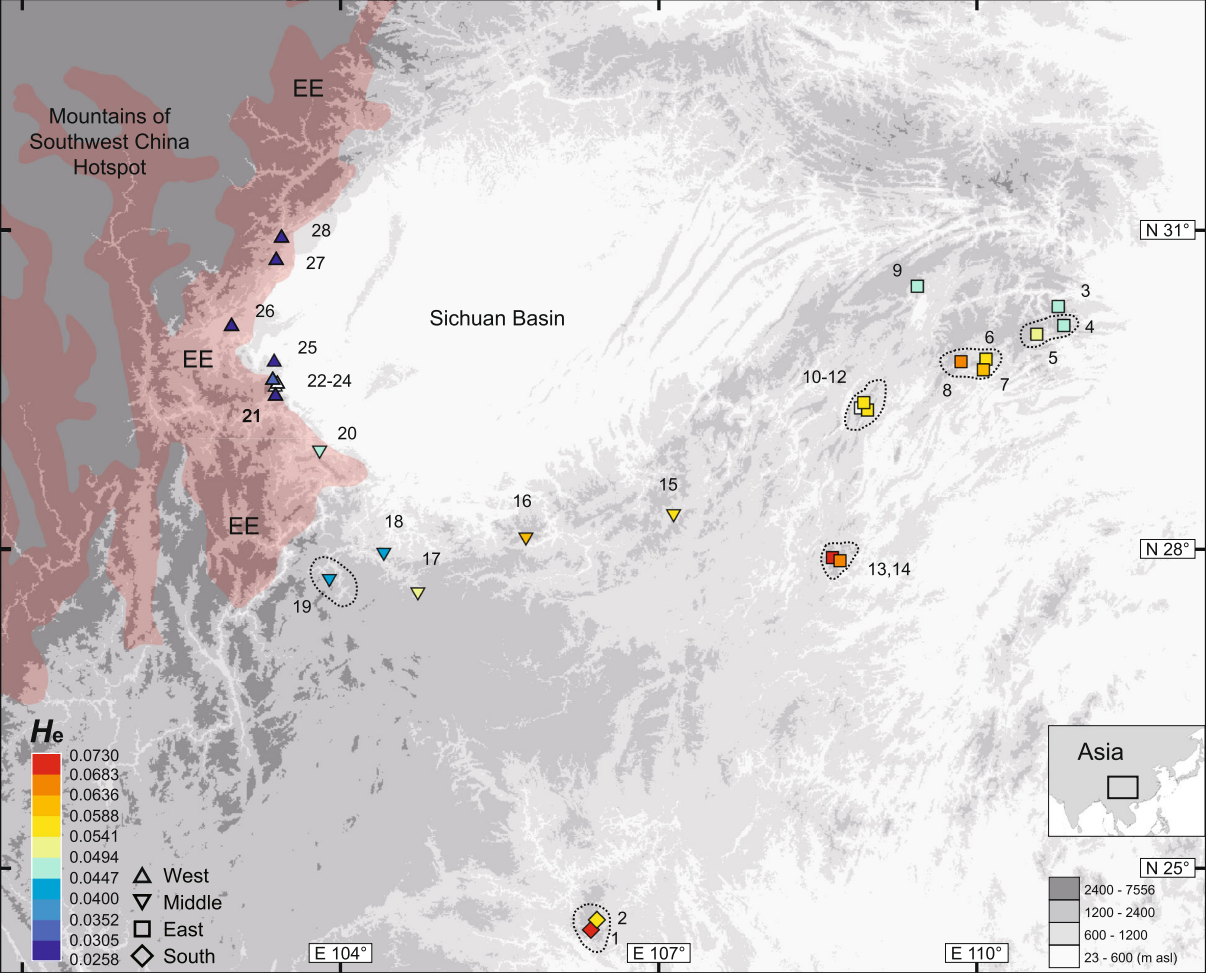
Sampling sites of *Leptobrachium boringii*, the eastern part (red) of the Mountains of Southwest China biodiversity hotspot, and geographic pattern of expected heterozygosity (*H*_e_) estimated from the 10,869-SNP data. Most known local distribution ranges are smaller than the sampling site symbols on the map. The others may cover wider areas enclosed with dotted lines. All the reported ranges were sampled. White indicates SNP data not collected. EE: the eastern edge of the hotspot. The map was created using DIVA-GIS version 7.5.0.0 (http://www.diva-gis.org/)

The Sichuan Basin and surrounding mountains (Fig. 1) in southwestern China constitute one such region. The topography of these basin-mountain systems was mainly shaped by tectonic activity before the Late Pliocene (3.6–2.6 million years ago; mya) [22–25]. During the Pleistocene, this region remained unglaciated except for a few marginal high-elevation areas in the west [26, 27]. Within this region, many organisms are confined to the mountain areas, resulting in a “ring” of higher species richness around the basin for some major lineages, including vascular plants, mammals, passeriform birds, and amphibians [28–31]. Some of these species are distributed continuously and may have a ring-shaped divergent pattern [32–37]. Many others have disjunct distributions ranging from much of the surrounding mountains to one area in the mountains, with most species distributions somewhere between these two extremes [38–40], which can potentially be explained by a dynamic history involving recurrent range fragmentation and contractions. In the western part of the region, the disjunct distribution of a variety of plant and animal species overlaps with the eastern edge of the Mountains of Southwest China hotspot [41–48], contributing to the biodiversity of the global hotspot and implying a shared long-term refugium [38, 39, 49–53]. Relevant studies that provided both species history inferences and multilocus estimates of genetic diversity distributions for such taxa are rare. They have focused on flowering plants, revealing local persistence through Quaternary climatic cycles and the possibility of extensive pollen-mediated gene flow while detecting, in hotspot edge areas, not necessarily unique or high genetic variation [39, 45, 54, 55]. Further case studies from other major evolutionary lineages would be beneficial.

The megophryid Emei mustache toad, *Leptobrachium boringii* (Liu, 1945), has a typical disjunct distribution in the mountains at the edges of the basin and hotspot (Fig. 1). This species occurs along the southern-half edge of the basin and in a small insular southern range, forming the northwestern range limit of the genus [56]. The toad inhabits mountain valleys covered by broad-leaved forest at elevations of 600 to 1700 m, breeds in permanent streams from February to March, and remains terrestrial outside the breeding season; additionally, the tadpoles usually take 3 years to reach metamorphosis [57, 58]. The “mustaches” are 10 to 16 maxillary keratinized nuptial spines used in male-male combat, and such structures occur only in *L. boringii* and close relatives among extant amphibians [58, 59]. Overcollecting for pet trade and habitat reduction are the main threats to this endangered (EN) species [60]. Previous studies with limited *L. boringii* sampling suggested pre-Quaternary divergences among major lineages [61] and a close relationship between the middle and eastern populations [62]. A further elaboration of the evolutionary processes and distribution of genetic diversity will contribute to the conservation of this species and may provide insights into the factors behind the similar disjunct distributions frequently observed in other frogs (e.g. *Xenophrys omeimontis*, *Rhacophorus omeimontis*, *Babina daunchina*, *Bombina lichuanensis*, [63, 64]).

In this study, we examine the distributional history and genetic diversity of the Emei mustache toad, focusing on two questions: (1) is the distribution highly dynamic over time, and what are the effects of range dynamics, and (2) does the eastern edge of the Mountains of Southwest China hotspot provide a long-term refugium harboring high genetic variation? We obtained sequence data for two mitochondrial loci and genome-wide single nucleotide polymorphism (SNP) data for samples from all of the corresponding known ranges. By combining time-calibrated gene tree, genetic diversity, genetic clustering, population splitting and mixing inference, and paleoclimatic suitability modeling, we inferred considerable range dynamics underlying the genetic and distribution patterns and a long-term refugium in the hotspot edge area, with the lowest genetic variability despite immigration. In addition, we demonstrated significant discordance between variability estimates from genome-wide SNPs and mitochondrial DNA sequences.

## Results

### Molecular data

The mitochondrial DNA dataset contained 1831 sites and 83 haplotypes after removing alignment gaps in the control region. Among the ingroup members, 282 sites were variable, and 227 sites were parsimony informative. Haplotype designation and GenBank accession numbers are given in Table S1. The genome-wide SNP main dataset contained 10,869 SNPs, from 7045 to 10,423 with a mean of 9305 SNPs (85.6%) for the ingroup and 6986 and 5680 SNPs for the two outgroup individuals. The 4-individual dataset with no missing loci included 16,888 SNPs. The Sequence Read Archive (SRA) accession numbers under BioProject PRJNA551927 are presented in Table S1. These three datasets are available on Dryad (10.5061/dryad.t4b8gtj0c).

### Genetic structure and divergence dates

**Fig. 2:**
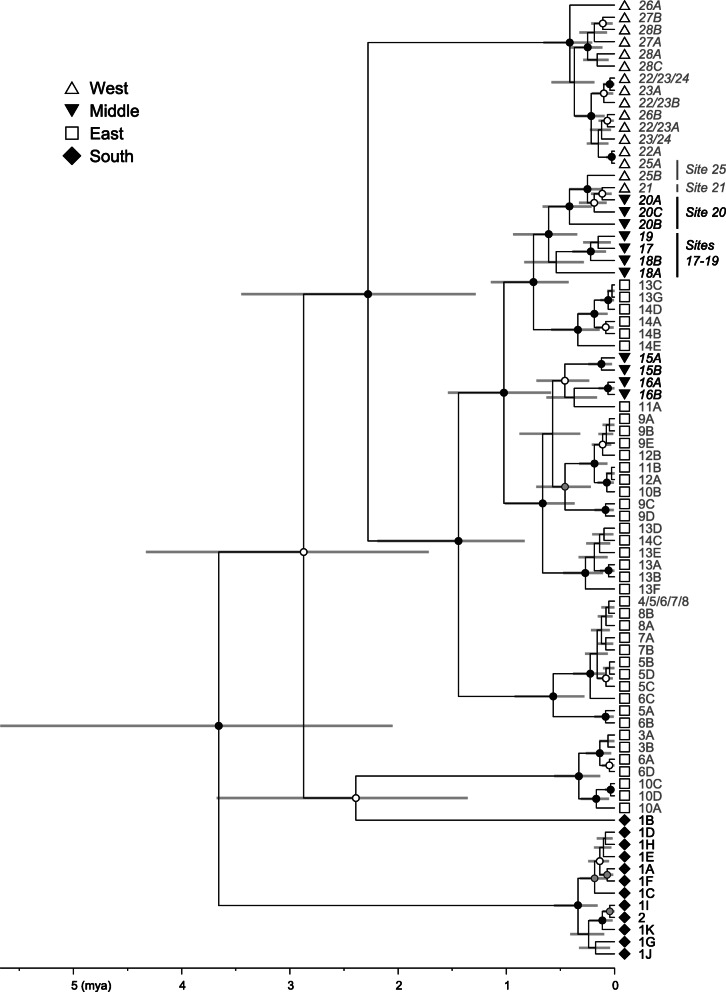
Time-calibrated mitochondrial gene tree of *Leptobrachium boringii* reconstructed with BEAST. Outgroups are not shown. Gray bars represent the 95% highest posterior densities for the age estimates. Nodes with Bayesian posterior probabilities ≥0.99, 0.95–0.98, and 0.80–0.94 are marked with black, gray, and open circles, respectively. Numbers appearing in haplotype names indicate sampling sites

For the BEAST analysis, a three-partition scheme was selected for the mitochondrial dataset: the protein-coding genes with the TrN + G model, tRNA genes with the K80 + I model, and control region with an HKY + I + G model. An adequate effective sample size of greater than 700 was achieved for each parameter [65], and the resulting topology was relatively well supported (Fig. 2). Haplotypes were rarely shared among sampling sites. Five major mitochondrial lineages that diverged at approximately 3.7 to 2.3 mya were detected, including four corresponding exclusively to regions South (sites 1 and 2), East (3–14), or West (21–28). In the other major lineage, haplotypes from the Middle region (sites 15–20) were nested within East clades, and sequential branching events from approximately 0.8 to 0.1 mya were inferred from East through Middle to West.

**Fig. 3:**
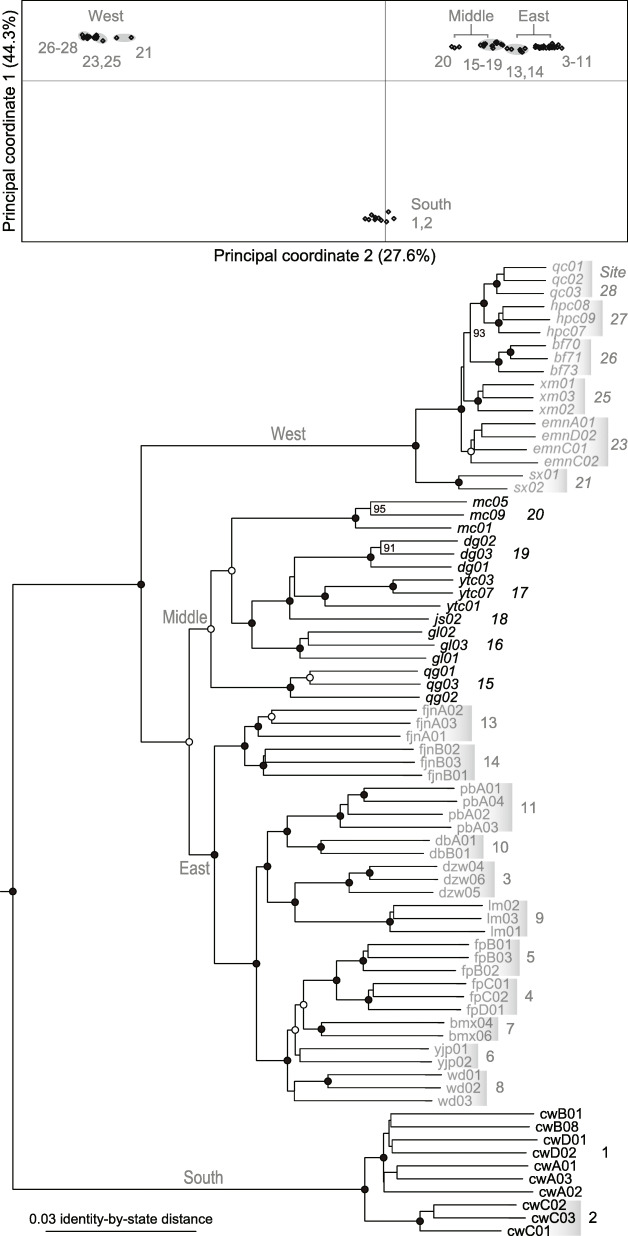
Principal coordinate plot and neighbor-joining tree (outgroups not shown) based on the SNP identity-by-state distance matrix for *Leptobrachium boringii* individuals. Numbers at nodes indicate bootstrap support values ≥90. Nodes with bootstrap support values of 100 and 97–99 are marked with closed and open circles, respectively. The names of the sampling sites correspond with those in Fig. 1. Outgroups are not shown

The first two principal coordinates obtained by PCoA jointly accounted for 71.9% of the observed genome-wide SNP variation among individuals (Fig. 3). Three discrete clusters corresponding to the South, East+Middle, and West regions were grouped. The latter two clusters were most closely related. Between them, samples from the most closely located sites 20 and 21 were also most closely related. The neighbor-joining tree (Fig. 3) could be summarized as (South,((East,Middle),West)), with individuals of each sampling site forming a unique cluster and an average bootstrap support of 97.2 for these clusters and their relationships. Sequential westward divergences were inferred among the Middle samples.

**Fig. 4:**
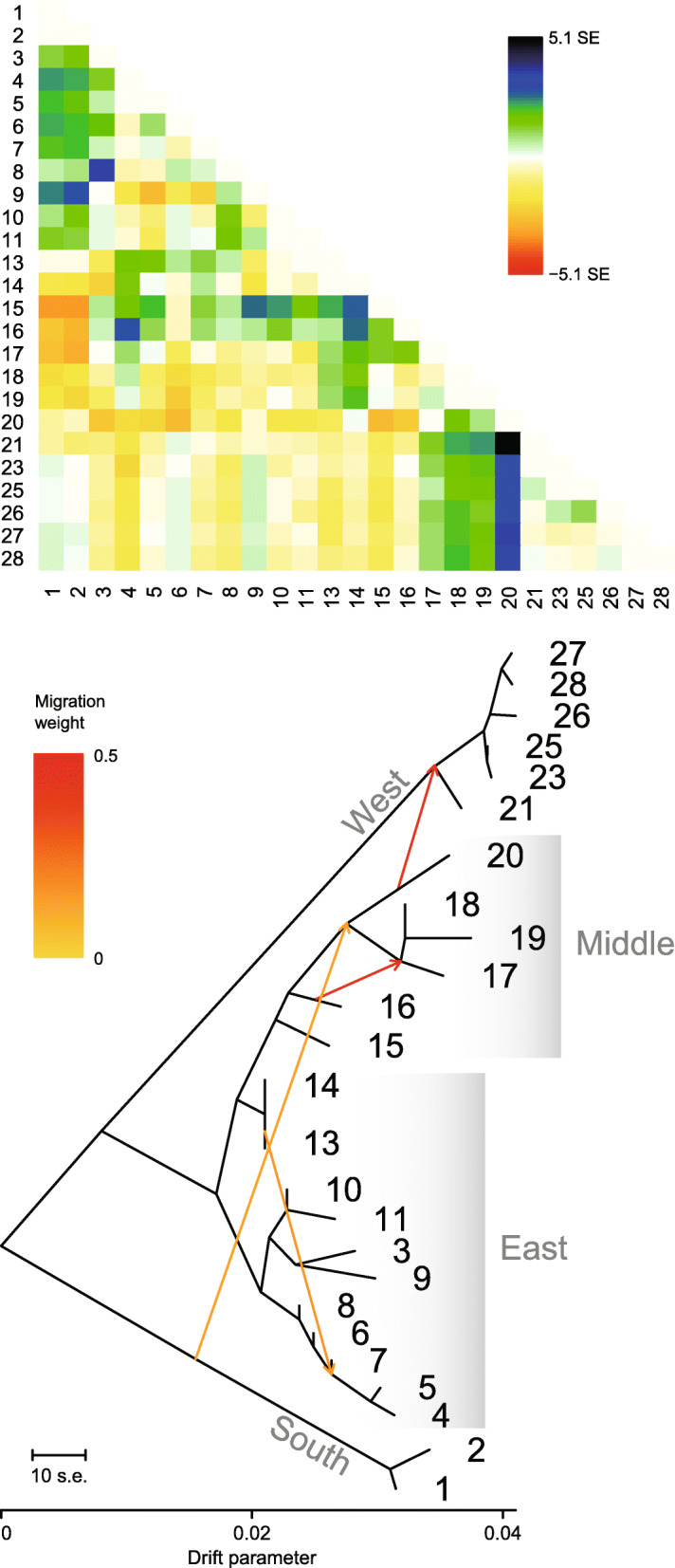
Residual fit (top) from the maximum-likelihood population tree without migration and the admixture graph (bottom) with four migration edges (arrows) inferred by TreeMix. The names of the sampling sites correspond with those in Fig. 1. Outgroups are not shown. Large residuals indicated potential candidates for admixture events. The weight of the migration edge represents the fraction of ancestry derived from the edge

Population relationships showing clear westward divergences and secondary contact were estimated by the TreeMix analysis of SNP data. Large residuals suggesting admixture between the Middle site 20 and the West sites, especially 21, were obtained when migrations were not allowed (Fig. 4). When migration edges were added, an edge with a weight of 0.476 from site 20 to the root of the West clade was found to most increase the likelihood. In addition, another three statistically significant edges (*P* < 0.05) involving some Middle, East, and South populations were added, with a resulting topology highly consistent with that of the neighbor-joining tree (Fig. 4).

### Genetic diversity

Various levels of regional genetic variation and a decline in variability along the path of sequential westward divergence were inferred from the main SNP dataset (Fig. 1, Table 1). The expected heterozygosity and mean proportion of heterozygous loci estimates were similar and strongly correlated (Pearson’s *r* = 0.972, *P* < 0.001, *n* = 25), with the highest values for some South and East populations and lowest ones across the West region. Average identity-by-state distances of 0.032, 0.076, 0.075, and 0.058 were obtained for the West, Middle, East, and South regions, respectively. When compared to the outgroup species, again, much lower variation was estimated for the West representative. From the 4-individual SNP dataset without missing loci, heterozygous loci proportions of 0.0616, 0.1462, 0.1614, and 0.1152 were obtained for West representative bf71, East representative fjnB02, and the two outgroups ms07 and yb01, respectively.

**Table 1:** Genetic diversity estimates for each sampling site of *Leptobrachium boringii*. *n*: number of individuals; *H*_e_: expected heterozygosity; $\overline{H}$: mean proportion of heterozygous loci across individuals; *h*: number of haplotypes; *Hd*: haplotype diversity; *π*: nucleotide diversity

Distribution region	Sampling site	10869 genome-wide SNPs			1831 bp mitochondrial DNA			
		*n*	*H*_e_	$\overline{H}$	*n*	*h*	*Hd*	*π*
South	1	7	0.0730	0.0618	22	11	0.931	0.0155
	2	3	0.0565	0.0557	9	1	0	0
East	3	3	0.0464	0.0445	8	2	0.429	0.0002
	4	3	0.0479	0.0391	5	1	0	0
	5	3	0.0517	0.0431	11	5	0.618	0.0050
	6	2	0.0567	0.0483	10	5	0.867	0.0241
	7	2	0.0599	0.0556	9	3	0.639	0.0005
	8	3	0.0649	0.0614	10	3	0.378	0.0002
	9	3	0.0449	0.0401	22	5	0.684	0.0021
	10	2	0.0560	0.0522	8	4	0.643	0.0108
	11	4	0.0555	0.0487	12	2	0.409	0.0022
	12	n/a	n/a	n/a	3	2	0.667	0.0015
	13	3	0.0679	0.0585	16	6	0.783	0.0074
	14	3	0.0704	0.0585	8	5	0.857	0.0068
Middle	15	3	0.0569	0.0510	15	2	0.343	0.0008
	16	3	0.0591	0.0504	12	2	0.167	0.0001
	17	3	0.0513	0.0452	8	1	0	0
	18	1	0.0432	0.0469	2	2	1	0.0088
	19	3	0.0430	0.0389	8	1	0	0
	20	3	0.0451	0.0408	12	3	0.621	0.0033
West	21	2	0.0300	0.0293	2	1	0	0
	22	n/a	n/a	n/a	6	4	0.800	0.0017
	23	4	0.0329	0.0275	28	5	0.632	0.0016
	24	n/a	n/a	n/a	8	2	0.250	0.0008
	25	3	0.0301	0.0262	7	2	0.286	0.0111
	26	3	0.0261	0.0258	11	2	0.436	0.0014
	27	3	0.0258	0.0240	21	2	0.095	0.0002
	28	3	0.0270	0.0253	8	3	0.464	0.0022

A similar variability pattern was not obviously represented in the mitochondrial diversity measures (Table 1). The nucleotide diversity and haplotype diversity were not correlated with the SNP expected heterozygosity (*P* > 0.05), and only a weak correlation was detected between the heterozygosity and haplotype number (Pearson’s *r* = 0. 474, *P* = 0.017, *n* = 25). For example, for the five single-haplotype sampling sites, expected heterozygosities of 0.0300–0.0565 were obtained, covering a 56.1% range of the estimates. In 24 sampling sites, both mitochondrial and SNP data were obtained for at least two individuals. Similarly, when only these 74 individuals were considered, all the three mitochondrial diversity measures (results not shown) were not correlated with the SNP expected heterozygosity (*P* > 0.05).

### Climatic suitability

**Fig. 5:**
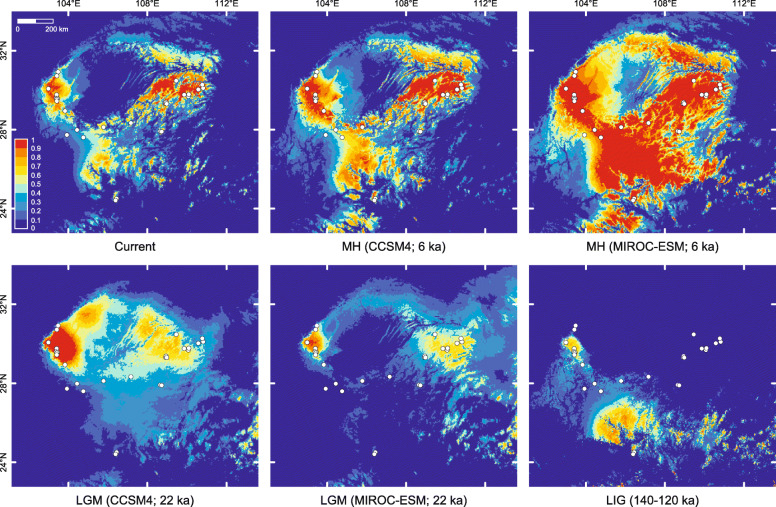
Climatic suitability for *Leptobrachium boringii* determined by Maxent modeling. Circles are both occurrence records and sampling localities. The maps were created in the R environment using the package raster version 2.8 (https://cran.r-project.org/web/packages/raster/index.html)

The inferred regional climatic suitability of *L. boringii* in different periods was not equally stable (Fig. 5). The Maxent model provided reasonable discrimination, with an average AUC of 0.955 (SD = 0.015). The three variables that provided the greatest contributions to model development were the mean temperature of warmest quarter (37%), isothermality (25%), and precipitation of warmest quarter (13.9%). The remaining variables, including precipitation of driest month, annual precipitation, mean temperature of driest quarter, and temperature annual range, provided 8.9, 6.8, 4.4, and 4% contributions, respectively. Around West sites 21–26, the potential LIG, LGM, and MH distributions overlap extensively with the eastern edge of the Mountains of Southwest China hotspot. In contrast, at the southern edge of the Sichuan Basin, mountains harboring mainly the Middle sites were inferred to be generally unsuitable during the LIG and LGM periods. Compared with the distributions in the current interglacial (present and MH) period, a more restricted LIG projection was obtained.

## Discussion

### Historical range dynamics and current disjunct distributions

In the mountains along the southern-half edge of the Sichuan Basin, our results indicate a Late Pliocene or early Pleistocene divergence between East and West *L. boringii*, much later westward dispersal establishing intermediate populations and causing secondary contact, and regionally varying climate stability. The East/West divergence was dated at 2.28 mya with a 95% highest posterior density of 3.45–1.28 mya (Fig. 2), overlapping with a previous estimate of 4.74–3.16 mya [61] and suggesting the long-term occupation of the two regions. Accordingly, a clear differentiation between the East and West samples was observed (Figs. 3 and 4). Westward dispersal occurring at approximately 0.8–0.1 mya was supported by the close relationship between the Middle and East samples, which agrees with the findings of Zheng et al. [62], and by the sequential westward divergences (Figs. 2, 3 and 4) accompanied by a significant loss of genome-wide SNP variation (Fig. 1, Table 1). The secondary contact is identified by the West mitochondrial DNA haplotypes nested within those of the western-most Middle site 20 (Fig. 2) and by a strong migration edge from site 20 to the West clade in the maximum-likelihood population tree inferred from the SNP data (Fig. 4). In addition to the pattern of secondary contact via dispersal after long-term isolation, the migration edges inferred within and between the East, Middle, and South regions suggest historical range changes. In line with this, local populations 4–8 are most closely related, and all exhibit a mitochondrial haplotype 4/5/6/7/8, implying recent admixture. It is likely that profound variations in regional climate stability, as exemplified by the modeled climatic suitability in the LIG, LGM, MH, and present-day periods (Fig. 5), have contributed to range dynamics and the current disjunct distribution of *L. boringii*. Nearly each part of the mountains surrounding the basin was inferred to be generally unsuitable in one or more periods. Meanwhile, distinct endemic genetic lineages such as those of sites 9, 13–16, 18, 20, and 26 (Fig. 2) support persistence in local refugia, which may be small given the complex mountain topography. We hence recommend caution in interpreting our climatic suitability results, and mainly use a general pattern implied by them together, recurrent range fragmentation and contraction, for hypothesis generating. Under suitable conditions, large range expansions might have not occurred in some refugial populations [8, 16]. This possibility and the recurrent range fragmentation and contraction caused by Quaternary climatic changes could lead to a largely disjunct distribution. Such a model provides a hypothesis for explaining scattered ranges and can be considered in future studies of the evolutionary histories of frogs and many other organisms isolated in the mountains surrounding the Sichuan Basin [38, 39, 48, 63, 64, 66, 67].

A substantially different modeled LIG distribution compared with those during the present interglacial period is not unique to the Emei mustache toad. Around the Sichuan Basin, such results have been commonly obtained for various animal [49, 50, 68–71] and plant groups [45, 55, 72, 73]. Because the LIG period at ~ 140–120 ka is relatively recent on an evolutionary scale, it is unlikely that the corresponding effects have been erased. In fact, a few studies have highlighted the significant roles of the LIG climate in shaping species histories [49, 69, 74, 75]. In theory, under global climatic cycles, profound climatic differences between the last and present interglacial periods should be limited to certain regions, which vary with the climatic variables of interest. An awareness of this relation may be beneficial, especially for examining the evolutionary history of narrow-ranged species and understanding regional biodiversity patterns.

The basal phylogenetic position of the southern population (Figs. 2 and 4) provides a clue to the early distributional history of the species. In the present distribution of *L. boringii*, the South region (Fig. 1) is most close to the distribution or proposed ancestral range of the close relatives of the species [15, 56, 62, 76–78]. It may be not far away from the ancestral range of the species and, as suggested by the high and unique genetic variation estimates (Fig. 3, Table 1), has probably served as a long-term refugium. One plausible scenario is that the eastern range was established through northward expansion no later than early Pleistocene, given that the West clade with a Late Pliocene to early Pleistocene origin was nested within eastern lineages according to our time-calibrated tree (Fig. 2). Then, the eastern population might have expanded to the western range by a route along mountains at the southern edge of the basin, which were reoccupied through the aforementioned, much latter westward dispersal.

### Spatial heterogeneity of the factors that influence high biodiversity within a hotspot

This study detects both long-term refugial and young immigrant (site 20) populations in the eastern edge of the Mountains of Southwest China hotspot. The molecular results suggest a Late Pliocene to early Pleistocene origin for the West clade endemic to the hotspot edge, in which the area around sites 21–24 and 26 has consistently been modeled to be suitable for *L. boringii* in the LIG, LGM, MH, and current periods. Western marginal refugia and areas of long-term persistence within or overlapping with this hotspot edge have been proposed for some mountain amphibian [37, 79], mammal [47], insect [50], and flowering plant species [38, 39, 52, 53, 80–82]. Additionally, gene flows from the east via mountains along the edges of the Sichuan Basin have recently been reported for different species [37, 48, 83]. Together with these findings, our results imply that the preservation of eastern taxa, facilitated by the continuous mountain topography surrounding the basin, contributes significantly to regional biodiversity.

In this hotspot, except for the southwestern finger, which overlaps the Nujiang Valley, the eastern edge is the only area that provides low-elevation habitat, say, below 1000 m. Most areas of the hotspot exceed 3000 m due to the rapid uplift of the Hengduan Mountain region mainly between the late Miocene and Late Pliocene [24, 84–87], which has been identified as a major driver of the diversification of hotspot flora by creating conditions favoring in situ speciation [88]. These areas are generally much higher than the mainland to the east and are not suitable for low-elevation species. Indeed, in addition to the aforementioned taxa, the eastern edge of the hotspot forms a western range limit for many other species [45, 54, 64, 89–91]; this is not surprising given that the biodiversity hotspots in temperate realms usually host a small proportion of endemics and numerous immigrant species, especially for mammals and birds [92, 93]. However, the pattern suggests that a certain contributor to biodiversity, preserving and receiving low-elevation elements in this case, can be significant for only a particular part of a hotspot. In noting such heterogeneity, we do not overlook the fact that the eastern edge exhibits highly complex geological structures that facilitate diversification [71] and harbors endemics at different elevations [94, 95]. Hotspots are critical for global biodiversity conservation [92]. Further studies on the heterogeneous nature of each hotspot may aid in conservation planning.

### Genome-wide SNP versus mitochondrial DNA diversity estimates

In addition to implying that long-term occupation does not necessarily correlate with large genetic variation at the western edge of the Sichuan Basin (Figs. 1, 2 and 5), our results provide an instance of discordant variation estimates using genome-wide SNPs and mitochondrial DNA sequences. Among the mitochondrial results, only the haplotype numbers are weakly correlated with the SNP expected heterozygosities (Table 1). Relatively reliable estimates of heterozygosity can be obtained using a large number of SNP loci from even a small number of individuals [20]. Compared with such estimates, mitochondrial DNA diversity is more susceptible to genetic drift and selective sweeps due to the smaller effective population size and lack of recombination. Moreover, for the same reason and because of possible sex-biased dispersal, population admixture may not have left proportional immigrant mitochondrial alleles. This uncertainty can also lead to discordant estimates from the two types of data. A previous study of this species has reported genetic parameters consistent with female-biased dispersal [96]. In the West region, site 25 was unique for having mitochondrial haplotype samples of both local and immigrant origins (Fig. 2) and an estimated nucleotide diversity of 0.0111. This value was at least five times larger than those of the other West sites and was higher than estimates for most sites in the other regions. However, the SNP heterozygosity estimates for West sites were similar and much lower than those in all the other regions. Given the high genetic homogeneity revealed by a substantially smaller average identity-by-state distance (0.032) than that in other regions (0.058–0.076), we suggest considering a bottleneck in addition to evolutionary stasis and other scenarios in an effort to explain the low variability found in western *L. boringii*.

Different RADseq and mitochondrial variations associated with admixture have been reported in other taxa [97–99]. The discordance detected in the present work is relevant to the advantage of using a large number of loci to estimate levels of genetic variation [20, 21]; notably, different estimates from genome-wide loci and mitochondrial DNA can be obtained for populations that experienced admixture. An awareness of this possibility may facilitate the integration of results from different sources of data.

## Conclusions

A scenario of dispersal after long-term isolation can explain much of the spatial distribution of genetic variation in the Emei mustache toad, yet long-term persistence and secondary contact do not guarantee high variability. For many other taxa isolated in the mountains surrounding the Sichuan Basin, a model that combines recurrent contractions caused by Quaternary climatic changes and some failed expansions under suitable conditions for explaining scattered ranges can be considered when examining their evolutionary histories. More generally, this study provides insights into the heterogeneous nature of biodiversity hotspots and the discordant variability estimates using genome-wide and mitochondrial data.

## Methods

### Sampling

A total of 301 individuals, including 289 tadpoles, from 28 sampling sites representing all the known ranges of *Leptobrachium boringii* were used as the ingroup. The congeners *L. liui* and *L. leishanense*, with two tadpole samples each, were used as outgroups according to the current phylogenetic hypotheses [62, 76–78]. Sequences of two mitochondrial DNA fragments were obtained for all samples, and genome-wide SNPs were obtained for a representative subset of 77 individuals. Detailed sampling information is provided in Table S1.

### Mitochondrial DNA data

The 745-bp *nad1* fragment consisted of partial tRNA-Leu (16 bp) and NADH dehydrogenase subunit 1 genes; the *D-loop* fragment of ~ 1080–1350 bp in length contained regions of the cytochrome b gene (78 bp) and a control region with the tRNA-Thr, tRNA-Pro, and tRNA-Trp genes between them. For the *nad1* fragment, the primers Leu-1 L and Leu-5 L from Zheng et al. [62] and ND1-6H from Zheng et al. [15] were used in PCR and Sanger sequencing (ABI 3730). Sequences of this fragment for 19 individuals were also obtained from these two previous studies. The primers for the *D-loop* fragment were newly designed in this study: Vcb-1 L (5′-ATCggCggAC AACCTgTCgA AgACC-3′), Vcb-3 L (5′-CAACCTgTCg AAgACCCCTA CgT-3′), Vcr-2H (5′-gTggCTgATC CACCggAAgg TAAg-3′), and Vcr-4H (5′-gCTgATCCAC CggAAggTAA gATC-3′). Sequences of the light-strand encoded tRNA-Pro gene were converted into complementary strand sequences. Alignment was conducted with ClustalX version 2.1 [100] and visually checked.

### Mitochondrial diversity and time-calibrated gene tree

The *nad1* and *D-loop* fragments were concatenated for analysis. The nucleotide diversity and haplotype diversity were estimated for each local population using DnaSP version 6.10.01 [101]. To examine the population relationships and the dates of divergence events, a time-calibrated tree was reconstructed in a Bayesian framework with BEAST version 2.5.1 [102]. The best-fitting combination of nucleotide partitions and substitution models was determined with PartitionFinder version 2.1.1 [103] using the Bayesian information criterion. To avoid potential over-parameterization of the intraspecific analysis, only three data blocks were defined for evaluation: the protein-coding genes, tRNA genes, and control region. The protein-coding gene block contained mainly the *nad1* gene sequence. Because suitable fossil calibrations were not available, an evolutionary rate of 0.69 ± 0.3% divergence per million years per lineage commonly used for the anuran *nad1* gene was used to calibrate the tree [15, 104–106]. This approach required the protein-coding gene block to be constrained as one partition in analysis. The uncorrelated lognormal model was used in the relaxed clock method. The calibrated Yule model was specified for the tree prior. The uncorrelated lognormal relaxed clock mean (ucldMean) was constrained between 0.0039 and 0.0099 substitutions per site per million years, and a normal distribution was applied (mean 0.0069, sigma 0.0015). Four independent runs of 20 million generations each were performed to avoid local optima, and a sampling frequency of 2000 generations was applied. The performance of the runs was evaluated using Tracer version 1.6 [107] to ensure that the chains were converging and mixing sufficiently, and the final 90% of samples for each of the four runs were combined to produce a maximum clade credibility tree.

### Restriction site-associated DNA sequencing and SNP data

Genome-wide SNP data from RADseq loci were obtained for 75 ingroup representatives from 25 sampling sites (one to seven individuals per site, mostly three) and two outgroup individuals using the SLAF-seq [108] approach. Genomic DNA (~ 0.15 μg per sample) was incubated at 37 °C with the *Mse*I restriction enzyme, T4 DNA ligase, ATP, and *Mse*I adaptor containing barcode information. The restriction ligation was heat inactivated at 65 °C. The product was digested with the restriction enzyme *Hae*II at 37 °C and subsequently purified using Agencourt AMPure XP beads (Beckman Coulter). PCR was performed using the purified product and Phusion Master Mix (New England Biolabs) with universal and index primers. The PCR products were purified and pooled and then electrophoresed on 2% agarose gel. Fragments of 425–450 bp in size (with indexes and adaptors) were isolated, purified and diluted for sequencing using the PE150 Illumina Hiseq 2000 platform.

Raw reads were checked for quality using FastQC version 0.11.7 [109]. Data cleaning was conducted using the process_radtags program in Stacks version 2.0b [110], discarding reads with an uncalled base or an average Phred33 score of < 10 within a 22-bp sliding window. A de novo assembly of loci was conducted using the denovo_map.pl pipeline in Stacks. In a preliminary analysis of a representative subset of 32 individuals, the minimum number of reads required to form an allele, number of mismatches allowed between alleles, and number of mismatches allowed between loci during the construction of the catalog in Stacks were determined as 3, 4, and 5, respectively. These values maximized the number of polymorphic loci found in 80% of the individuals [111]. Through this pipeline, biallelic SNPs present in at least 80% of individuals with a minor allele frequency greater than 1%, one random SNP per locus, were obtained for further filtering using VCFtools version 0.1.17 [112]. The final main dataset was composed of SNPs with a mean read depth across individuals greater than 6 and below 50 to avoid the potential effects of sequencing errors and repetitive regions. In addition, keeping all settings equal except for allowing no missing data, a 4-individual dataset was generated to compare the genetic diversity of two ingroup representatives and two outgroup individuals.

### Genetic diversity and structure analyses of SNPs

The expected heterozygosity for each sampling site was estimated using Arlequin version 3.5.2.2 [113]. In addition, the heterozygosity for each individual was measured by the proportion of heterozygous loci using the --het option in VCFtools and then averaged across individuals for each sampling site. Using SPSS version 12.0, various diversity estimates were first checked for normality with the Kolmogorov-Smirnov test, and then a Pearson or Spearman correlation test was performed for comparison.

An identity-by-state distance matrix between individuals was constructed in PLINK version 1.9 [114] and used for principal coordinates analysis (PCoA) and neighbor-joining tree building. PCoA was conducted using GenAlEx version 6.502 [115]. A neighbor-joining tree was constructed using the Neighbor program in PHYLIP version 3.695 [116], with 500 bootstrap replicates to evaluate the nodal support. A Perl script VCFbootstrap.pl (10.5061/dryad.t4b8gtj0c) was written to generate the 500 bootstrap SNP datasets in VCF format. At the population level, a maximum-likelihood inference of splitting and mixing from the allele frequency was conducted using TreeMix version 1.13 [117] to assess the diversification and potential secondary contacts. The allele frequency at the sampling site level was generated by PLINK. Models allowing zero to five migration events were explored in separate TreeMix analyses, and the results were compared.

### Climatic suitability

As an approach to assess range stability, the last interglacial (LIG), last glacial maximum (LGM), and mid-Holocene (MH) potential distributions were modeled for comparison. Climate reconstructions for these periods are frequently used examples of Quaternary climate fluctuations. The analyses were conducted at a spatial extent of 21.5–34°N and 100–114°E, extending not substantially from the Sichuan Basin and surrounding mountains and the range of *L. boringii*.

Ecological niche modeling was conducted using Maxent version 3.4.1 [118, 119]. Nineteen current bioclimatic variables with a resolution of 2.5 arc-minutes were obtained from the WorldClim database [120]. The occurrence data containing 28 records, with no more than one occurrence per grid cell, were derived from our field surveys (10.5061/dryad.t4b8gtj0c). For highly correlated temperature/precipitation variable pairs (Pearson, |*r*| > 0.8), the variables with smaller contributions according to jackknife analysis of variable importance to model development were excluded [118, 121, 122]. Seven variables were retained: isothermality, temperature annual range, mean temperature of driest quarter, mean temperature of warmest quarter, annual precipitation, precipitation of driest month, and precipitation of warmest quarter. In a Maxent analysis, we ran models with 10 bootstrap replicates by randomly assigning occurrence records to the training (75%) and testing (25%) data sets. The replicates were used as proxy models to develop consensus-based ensemble forecasts [123], and the mean suitability was output in logistic format [124]. Model performance was assessed using the area under the receiver operating curve (AUC).

The niche model was projected onto paleoclimate reconstructions in WorldClim with the same resolution of 2.5 arc-minutes. The resolution of the LIG (~ 140–120 thousand years ago; ka) layers was changed from 30 arc-seconds to 2.5 arc-minutes through averaging [125]. For LGM (22 ka) and MH (6 ka), taking the uncertainty stemming from different global climate models into consideration, the commonly used reconstructions by the Community Climate System Model (CCSM4, [126]) and the Model for Interdisciplinary Research on Climate (MIROC-ESM, [127]) were adopted.

## Supplementary information

**Additional file 1: Table S1.** Sample information and haplotype designation. (XLS 87 kb)

## Data Availability

Mitochondrial DNA sequences: GenBank accessions KP054478–KP054954 and MK164282–MK164395. RADseq raw reads: Sequence Read Archive accessions SRR9645625–SRR9645701 under BioProject PRJNA551927. The Perl script VCFbootstrap.pl, occurrence data for ecological niche modeling, mitochondrial DNA dataset, main SNP dataset, and 4-individual SNP dataset are available on Dryad (10.5061/dryad.t4b8gtj0c).

## Abbreviations

ATP: Adenosine triphosphate
AUC: Area under the receiver operating curve
CCSM: Community Climate System Model
LGM: Last glacial maximum
LIG: Last interglacial
MH: Mid-Holocene
MIROC: Model for Interdisciplinary Research on Climate
mtDNA: Mitochondrial deoxyribonucleic acid
PCoA: Principal coordinates analysis
PCR: Polymerase chain reaction
RADseq: Restriction-site associated deoxyribonucleic acid
SLAF-seq: Specific-locus amplified fragment sequencing
SNP: Single nucleotide polymorphism
VCF: Variant call format
